# A Randomized Clinical Trial of Nutrition Education for Improvement of Diet Quality and Inflammation in Iranian Obese Women

**DOI:** 10.1155/2014/605782

**Published:** 2014-10-02

**Authors:** Majid Mohammadshahi, Fatemeh Haidari, Majid Karandish, Sara Ebrahimi, Mohammad-Hosein Haghighizadeh

**Affiliations:** ^1^Hyperlipidemia Research Center, Ahvaz Jundishapur University of Medical Sciences, Ahvaz 61357-15794, Iran; ^2^Nutrition and Metabolic Diseases Research Center, Ahvaz Jundishapur University of Medical Sciences, Ahvaz 61357-15794, Iran; ^3^Department of Nutritional Science, Arvand International Division of Ahvaz Jundishapur University of Medical Sciences, Ahvaz 61357-15794, Iran; ^4^School of Health, Ahvaz Jundishapur University of Medical Sciences, Ahvaz 61357-15794, Iran

## Abstract

*Background*. Obesity is considered as a low grade inflammation condition. The aim of this study was to investigate the effect of nutritional education on diet quality and biomarkers of inflammation in Iranian obese women. *Method*. Sixty obese women voluntarily participated in this randomized clinical trial and were randomly assigned to intervention or control group (*n* = 30). Intervention group was instructed to attend nutrition education sessions (1 hr/wk, for 3 months) in small groups. Diet quality scores were measured by Healthy Eating Index (HEI). Anthropometric indices and serum concentration of hs-CRP, TNF-*α*, and adiponectin were measured at the baseline and end of the intervention. *Results*. There were no significant differences in anthropometric indices of participants between the two groups at the end of intervention (*P* > 0.05). However, the total HEI score was significantly higher in the educated group compared to the control group after intervention (*P* < 0.05). The educated group also showed significant lower concentration of TNF-*α* and hs-CRP and higher levels of adiponectin than the control group at the end of study (*P* < 0.05). *Conclusions*. Our results provide limited evidence that higher dietary quality contributes to reduced inflammation in obese women. This effect could be independent of the weight loss.

## 1. Background

The link between unhealthy eating habits and obesity is well established [1–4]. Since human diets contain many components that may work synergistically to prevent or promote disease, assessing that diet quality may be an informative strategy when studying the relation between nutrition and obesity [5–8].

Indexes of dietary quality have been developed to address this need in nutrition research. Two of these, the Healthy Eating Index (HEI) and the Diet Quality Index (DQI), were developed to measure adherence to dietary guidelines and were shown to adequately measure overall diet quality [9, 10]. The HEI measures adherence to the Food Guide Pyramid developed in the mid-1990s, whereas the DQI reflects a person's adherence to the Diet and Health recommendations of the National Academy of Sciences [11].

Obesity is a low-grade inflammatory condition; however, limited studies to date have considered the overall quality of the diet and its relation to obesity-induced inflammation; these have been mainly limited to the cross-sectional studies [1, 12, 13]. According to findings from the National Health and Nutrition Examination Survey III, the HEI score was inversely associated with C-reactive protein (CRP) concentration, which was largely attributed to total grain consumption [13]. Fung et al., however, found no association between HEI score and various markers of inflammation and endothelial dysfunction [14]. Fargnoli et al. showed that adherence to a healthier diet, as reflected by a higher HEI score, is associated with higher plasma adiponectin and lower plasma resistin, CRP, and E-selectin levels independent of obesity and lifestyle factors [2]. Thus, dietary quality may be inversely associated with inflammatory conditions independent of BMI [12].

Although concentrations of inflammatory markers and adipokines were previously associated with modifiable lifestyle factors (or dietary patterns) in cross-sectional studies, diet quality has not been previously studied in relation to proinflammatory factors and adiponectin during nutritional intervention. According to Tehran Lipid and Glucose Study (TLGS), the diet quality of most people in Tehran, Iran, (74%) needs improvement and thus nutritional education to improve diet quality is recommended [15].

The purpose of the study reported here was to evaluate if nutrition education could improve diet quality, as measured by HEI score, and thereby it would be independent of weight loss associated with improving of inflammatory markers (hs-CRP and TNF-*α*) and adiponectin concentration.

## 2. Methods

### 2.1. Study Setting and Design

This study was designed as a randomized clinical trial to investigate the effects of nutritional education for 3 months on the improvement of HEI scores and serum levels of inflammatory markers in obese women. At first, a call for participating in the study was provided and the aim of the study was described completely to who attended to the Health and Nutrition Clinic of Ahvaz, Iran. Then regarding the inclusion and exclusion criteria, subjects were selected. Inclusion criteria were women with body mass index (BMI) ≥30, having an initial HEI score <80, under conditions of weight stability for at least 3 months, and subject satisfaction. Exclusion criteria included chronic disease (diabetes, cardiovascular disease, cancer, arthritis, lung disease, asthma, or serious allergies), being active in exercise or weight reduction programs, taking weight loss medications, taking drugs that are known to affect the immune system, including nonsteroidal anti-inflammatory medications and corticosteroids, consumption of nutritional supplements at least 6 months before sampling, alcohol consumption, vegan diet, smoking, and lack of subject follow-up (being absent in 2 or more education sessions). We also excluded under and over reporters of energy intakes [15].

### 2.2. Study Population, Sample Size, and Sampling

Finally, sixty obese women, aged 20–45 y, who had volunteered, were randomly divided into two groups (education group: *n* = 30 and noneducation group: *n* = 30) using a table of random numbers with block size not revealed to the investigators. Those randomized to the intervention (education) group were instructed to attend nutrition education sessions (1 hr/wk for 3 months, 12 sessions) in small groups (6 persons per session) conducted by a registered dietitian. All subjects in this group received the same information over the course of 3 months of participation. Each 1 hr session included an oral presentation on nutrition information relevant to healthy diet and HEI recommendations. In this study, the nutrition education program was based on the modified My Plate (regarding to Iranian food culture) and food pyramid guideline. All participants in the education group were also provided with a designated booklet related to nutrition education. The control group in this study did not receive the nutrition education program and they were educated for food recording only.

### 2.3. Data Collection and Procedure

In this study, all participants were requested not to change their physical activity habits during the study. At baseline and the end of the study, anthropometric measurements (weight, height, waist circumference (WC), and hip circumference (HC)) were measured and then waist-to-hip ratio (WHR) was determined. Body mass index (BMI) was calculated from measured height and weight. Participants' body fat percent was also measured by body state set (Quad Scan 4000).

Dietary intakes were assessed by the seven consecutive days' food record. On the basis of 7 days' food record, each HEI component score was calculated for each individual. The HEI total score was also calculated as the sum of these scores over the 12 components. Then, for each component score and for the total score, the mean score was taken. Briefly, the HEI-2005 comprises 12 components, which are scored on a scale from 0 to maximum (*M*), where *M* is 5, 10, or 20 according to the component. Thus, the composite HEI score can potentially range from a minimum of zero to a maximum score of 100, with 100 points referring to perfect diet quality and lower results indicating larger deviations from the recommended intakes [7]. For each individual and each component, the ratio of the reported intake of food group (relevant to the HEI component considered) to the reported energy intake was also calculated. Then, the mean of these ratios over the individuals was taken. All of the dietary and anthropometric data were collected by an expert registered dietitian. In this study, a modified Nutritionist IV program was used to estimate dietary intake of participants.

### 2.4. Biochemical Analysis

At baseline and the end of the study, fasting blood samples were also collected from all the participants, and sera were separated for biochemical analyses. Serum concentrations of hs-CRP, TNF-*α*, and adiponectin were measured using a commercially available enzyme-linked immunosorbent assays (ELISA) method (Labor Diagnostika Nord for hs-CRP and Orgenium laboratories-Finland for TNF-*α* and adiponectin). All assays were performed according to the manufacturer's instructions.

### 2.5. Statistical Analysis

All statistical analyses were performed with Statistical Package for Social Sciences (SPSS Inc., Chicago, IL, USA) Program version 18 for windows. At first, normal distribution of all variables was checked with the Kolmogorov-Smirnov test. Group variable means were compared with each other using both independent sample *t*-test and ANCOVA in the adjusted models, which were adjusted for confounders (age, weight, and energy intake). The end values of each variable were also compared with the baseline values using paired sample *t*-test. The differences with *P* values <0.05 were considered as significant.

### 2.6. Ethnic

The study was approved by and performed under the guidelines of the Research Ethics Committee of Ahvaz Jundishapur University of Medical Sciences, Iran (ETH-9115). A written consent was also obtained from all the participants.

## 3. Results

In this study, 60 participants with 34.15 ± 5.34 years old were included in the study. Sociodemographic and anthropometrics characteristics of the study participants at baseline and the end of the study are described in Table 1. There were no significant differences in baseline and end of study measures of participants (age, income, education, physical activity, weight, BMI, WC, HC, WHR, and fat percent) between the two groups. The anthropometric indices did not also decrease within groups at the end of study when compared to the baseline values.

Dietary intakes of the study participants are presented in Table 2. Total carbohydrate and total fat intake of participants decreased at the end of the study compared to the educated and the noneducated groups at baseline (*P* < 0.05). At the end of study, dietary intakes of total fat also decreased significantly in the educated group when compared to the control group (*P* = 0.000). This result remained significant after adjustment for confounding variables (age and energy intake).

As shown in Table 3, the mean total HEI score was 60.58 ± 6.31 in educated group and 62.05 ± 5.7 in noneducated group at the baseline. Therefore, the group differences in baseline total HEI scores were not significant in this respect. Mean component scores were not significantly different between the two groups at the baseline. At the end of study, the total fruits, total vegetables, dark green and orange vegetables and legumes, whole grains, milk, meat and beans, saturated fats, sodium, and SoFAAS (calories from solid fats, alcoholic beverages, and added Sugars) component scores and consequently the total HEI score were significantly higher in the educated group compared to the control (*P* < 0.05). These results remained significant after adjustment for confounders. After 3 months of nutrition education, the mean total HEI score was also significantly increased in the educated group, compared to baseline values (83.34 ± 5.12 versus 60.58 ± 6.31, *P* = 0.000).

The relative intake of each HEI component to the energy intake is presented in Table 4. The average intake of total fruits, whole fruits, total vegetables, dark green and orange vegetables and legumes, total grains, whole grains, and milk per 1,000 kcal significantly increased after intervention in the educated group compared to baseline intakes (*P* < 0.05). Furthermore, the relative intake of oils, saturated fat, and sodium components significantly decreased in this group (*P* < 0.05). After intervention, the comparison between two groups also showed that the relative intake of total fruits, whole fruits, total vegetables, dark green and orange vegetables and legumes, total grain, whole grains, milk, and meet and beans per 1,000 kcal was significantly higher in the educated group. In contrast, a significantly lower relative intake of oils, saturated fat, sodium, and SoFAAS per 1,000 kcal was observed in the educated group, compared to the control group, at the end of study (*P* < 0.05). These differences even remained significant after adjustment for confounders.

As presented in Table 5, significant differences were observed between the two groups regarding serum levels of inflammatory biomarkers at the end of intervention. Serum levels of TNF-*α* and hs-CRP were significantly lower in the educated group compared to the control group (*P* = 0.044 and *P* = 0.021, resp.). This significance remained even after adjustment for age, weight, and energy intake (*P* = 0.011 and *P* = 0.039, resp.). The educated group also showed significant higher adiponectin concentrations compared to the control group at the end of study (*P* = 0.035, in the adjusted models). Statistical analyses (within groups) also showed that the serum levels of TNF-*α* and hs-CRP significantly decreased and the mean concentration of adiponectin significantly increased after intervention in the educated group (*P* < 0.05).

## 4. Discussion

The results of this study showed that 3-month nutrition education improved diet quality, as measured by the HEI. Consequently, the improved diet quality was associated with the lower levels of hs-CRP and TNF-*α* and the higher levels of adiponectin in obese women (*P* < 0.05). This effect was independent of weight changes.

The relationship of diet quality to obesity has been inconsistent, and lack of association between diet quality and anthropometric indicators of obesity has also been reported in other studies [1, 7, 16–19]. In a cross-sectional study conducted by Boynton et al., the HEI scores were modestly correlated with BMI, yet not with percent body fat in postmenopausal women. However, individuals with lower BMI or lower percent body fat had more healthful diets, as measured by the DQI [1]. One possible reason for these discrepant results is that while the DQI generally emphasizes moderation, half of the HEI score concerns meeting or exceeding the recommended amount of grains, fruits, vegetables, meat, and milk. Thus, an individual who eats more in general receives a higher HEI score, which may explain why no clear associations between HEI and body composition were observed [1, 17, 18].

Although recent studies have shown an important role for inflammation in obesity, limited studies have evaluated the association between diet quality and circulating level of inflammatory markers with respect to obesity [20]. Since human diets contain many components that may work synergistically to prevent or promote disease, assessing diet quality may be informative [6]. The results from a crossover study that was conducted in healthy women have shown that, after adjustment for age and energy intake, women with the highest adherence to the HEI had 24% higher plasma adiponectin and 41% lower plasma CRP than did women with the lowest adherence to the HEI. Inverse association between the HEI and TNF-*α* was also reported, but it was not significant after adjustment for body mass index [2].

Some studies have examined the contribution of major dietary patterns to markers of systemic inflammation. Lopez-Garcia et al., in a crossover study, showed that the prudent pattern that is characterized by higher intakes of fruit, vegetables, legumes, fish, poultry, and whole grains is inversely associated with plasma concentrations of CRP. Contrary to this finding, the Western pattern that is characterized by higher intakes of red and processed meats, sweets, desserts, French fries, and refined grains showed a positive relation with CRP and interleukin 6 after adjustment for confounders [21]. The prudent pattern and the Western pattern in Lopez-Garcia et al. study were relatively comparable to perfect diet quality and imperfect diet quality in the present study, respectively. In another study, median plasma adiponectin concentrations were 23% higher in women who most closely followed a Mediterranean-type diet than in low adherers [22]. Similar results were also reported by others [14, 23]. Similarly, Esposito et al., in a 2-y randomized controlled trial, reported that a Mediterranean-type diet accompanied by increased physical activity significantly increased adiponectin concentrations in obese postmenopausal women, even after accounting for the decreased body weight associated with the intervention [24]. The results of this study are consistent with those of the above studies and extend them by showing that adherence to a healthier diet, which is achieved by nutrition education in the educated group, was associated with higher adiponectin concentrations and lower hs-CRP and TNF-*α* levels. Several recent studies have shown that fiber, antioxidant, flavonoids, folate, vitamin C, beta carotene, selenium, magnesium, and cupper, which was found mainly in the HEI components (fruits, vegetables, legumes, and whole grains), are positively associated with improved metabolic responses and have beneficial effects on markers of inflammation [25, 26]. However, since foods are not eaten in isolation, recent studies affirmed the importance of the overall dietary pattern or dietary quality, rather than that of specific food groups or nutrients, to inflammatory markers [22]. Although, in the present study, the relation between total HEI components scores and inflammatory markers was not assessed, individuals with higher total HEI scores had significantly higher adiponectin concentration and lower hs-CRP and TNF-*α* level (Table 6). In this study, it was attempted to account for confounders that were likely associated with concentration of inflammatory markers or consumption of a diet. However, the potential for remaining confounders by uncontrolled covariates was possible, and the present study was also limited by the small sample size, short duration of the intervention, and the self-report of food intake. Finally, the sample used in this randomized clinical trial does not represent a random sample of the Iran population; thus future studies with larger sample size are needed to identify these determinants.

## 5. Implications for Research and Practice

The results of this study support the hypothesis that beneficial effects of improved dietary quality with respect to obesity may be partially mediated by improvements in plasma concentrations of adiponectin and other biomarkers of systemic inflammation. This effect could be independent of weight loss and the other anthropometric variations. Future studies should extend these findings by investigating potential mechanisms underlying these relations and by examining whether prevention of the diabetes, atherosclerosis, and metabolic syndrome by diet quality improvements is mediated through changes in clinically important biomarkers, including adiponectin, hs-CRP, and TNF-*α* concentration.

## Figures and Tables

**Table 1:** Sociodemographic and anthropometric characteristics of the study groups at the baseline and end of study.

Variables	Educated group (*n* = 30)	Noneducated group (*n* = 30)	*P*1	*P*2
Age (y)	33.13 ± 5.60	35.13 ± 4.97	0.211	—
Income [*n* (%)]			0.516	0.306
Low	4 (13.33)	6 (19.98)		
Medium	22 (73.26)	19 (63.27)		
High	4 (13.33)	5 (16.65)		
Educational level [*n* (%)]			0.911	0.783
<6 years	1 (3.33)	0		
6–12 years	5 (16.65)	7 (23.31)		
>12 years	24 (79.92)	23 (76.59)		
Physical activity [*n* (%)]			0.852	0.771
Light	22 (73.26)	21 (69.93)		
Moderate	6 (19.98)	8 (26.64)		
Heavy	2 (6.66)	1 (3.33)		
Weight (kg)				
Baseline	91.1 ± 10.6	92.5 ± 14.6	0.672	0.581
End	89.8 ± 12.8	91.8 ± 13.5	0.533	0.671
*P*3	0.145	0.348		
BMI				
Baseline	34.9 ± 3.9	34.7 ± 5.07	0.877	0.676
End	34.3 ± 4.3	34.5 ± 5.11	0.852	0.845
*P*3	0.090	0.466		
WC (cm)				
Baseline	107.3 ± 10.8	107.6 ± 13.2	0.910	0.721
End	106.2 ± 8.3	108.2 ± 13.5	0.510	0.511
*P*3	0.161	0.416		
HC (cm)				
Baseline	110.2 ± 10.4	108.8 ± 10.1	0.593	0.804
End	109.5 ± 7.6	109 ± 9.9	0.812	0.973
*P*3	0.744	0.832		
WHR				
Baseline	0.97 ± 0.10	0.98 ± 0.06	0.655	0.690
End	0.97 ± 0.09	0.99 ± 0.07	0.417	0.553
*P*3	0.802	0.424		
Fat percent (%)				
Baseline	34.6 ± 6.3	33.5 ± 7.2	0.211	0.189
End	33.8 ± 6.6	33.4 ± 6.2	0.425	0.385
*P*3	0.253	0.837		

BMI: body mass index; WC: waist circumference; HC: hip circumference; and WHR: waist to hip ratio.

Data were expressed as mean ± SD or percentages.

*P*1 resulted from independent sample *t*-test or chi-squared test as appropriate; *P*2 resulted from ANCONA test (adjustment for age and energy intake); and *P*3 resulted from paired sample *t*-test.

**Table 2:** Dietary intakes of the study groups at the baseline and end of study.

Variables	Educated group (*n* = 30)	Noneducated group (*n* = 30)	*P*1	*P*2
Energy (Kcal)				
Baseline	2232 ± 593	2086 ± 320	0.086	0.105
End	2041 ± 315	1931 ± 249	0.140	0.246
*P*3	0.066	0.073		
Protein (gr)				
Baseline	86.8 ± 25.8	84.1 ± 16.4	0.632	0.483
End	81.4 ± 12.5	79.4 ± 14.2	0.620	0.909
*P*3	0.234	0.061		
Protein (%)				
Baseline	14.8 ± 2.3	16.0 ± 1.7	0.091	0.063
End	16.0 ± 1.4	16.5 ± 2.1	0.307	0.242
*P*3	0.324	0.651		
Carbohydrate (gr)				
Baseline	260.2 ± 78.5	219 ± 44.2	0.116	0.522
End	206.7 ± 40.7	196 ± 37.1	0.297	0.103
*P*3	**0.001**	**0.006**		
Carbohydrate (%)				
Baseline	44.5 ± 6.3	42.0 ± 5.5	0.071	0.132
End	40.4 ± 4.0	40.6 ± 5.8	0.202	0.731
*P*3	0.001	0.082		
Fat (gr)				
Baseline	109.4 ± 30.2	101.7 ± 18.4	0.240	0.407
End	73.3 ± 18.7	97 ± 15.7	**0.000**	**0.000**
*P*3	**0.000**	**0.045**		
Fat (%)				
Baseline	34.6 ± 6.3	33.5 ± 7.2	0.201	0.083
End	33.8 ± 6.6	33.4 ± 6.2	0.251	0.206
*P*3	0.132	0.282		

Data were expressed as mean ± SD.

*P*1 resulted from independent sample *t*-test; *P*2 resulted from ANCONA test in the adjusted models; and *P*3 resulted from paired sample *t*-test.

**Table 3:** Component score of HEI in the study groups at the baseline and end of study.

Components of HEI	Educated group (*n* = 30)	Noneducated group (*n* = 30)	*P*1	*P*2
Total fruit				
Baseline	3.10 ± 1.4	3.14 ± 1.09	0.898	0.322
End	4.91 ± 0.33	3.78 ± 1.17	**0.000**	**0.000**
*P*3	**0.000**	0.110		
Whole fruit				
Baseline	4.27 ± 0.85	4.34 ± 1.25	0.797	0.872
End	4.77 ± 0.69	4.77 ± 0.69	0.734	0.500
*P*3	**0.003 **	0.057		
Total vegetables				
Baseline	3.18 ± 1.14	3.44 ± 1.02	0.354	0.119
End	4.68 ± 0.52	3.28 ± 0.98	**0.000**	**0.000**
*P*3	**0.000**	0.400		
Dark green and orange vegetables and legumes				
Baseline	1.53 ± 1.41	1.64 ± 1.27	0.750	0.960
End	4.36 ± 0.86	1.64 ± 1.13	**0.000**	**0.000**
*P*3	**0.000**	0.598		
Total grain				
Baseline	4.92 ± 0.22	4.80 ± 0.35	0.802	0.792
End	4.94 ± 0.47	4.93 ± 0.33	0.858	0.702
*P*3	0.873	0.532		
Whole grain				
Baseline	0.4 ± 0.1	0.13 ± 0.07	0.036	0.021
End	3.1 ± 1.3	0.13 ± 0.49	**0.000**	**0.000**
*P*3	**0.000**	0.256		
Milk				
Baseline	0.89 ± 0.62	0.77 ± 0.34	0.344	0.099
End	5.52 ± 2.23	0.64 ± 0.53	**0.000**	**0.000**
*P*3	**0.000**	0.242		
Meat and beans				
Baseline	6.44 ± 1.95	6.36 ± 2.31	0.881	0.329
End	7.86 ± 2.03	5.9 ± 1.99	**0.000**	**0.000**
*P*3	**0.005**	0.237		
Oils				
Baseline	7.25 ± 1.81	7.60 ± 1.12	0.377	0.429
End	8.41 ± 2.25	8.32 ± 1.15	0.843	0.754
*P*3	**0.025**	0.032		
Saturated fat				
Baseline	7.38 ± 1.54	7.31 ± 1.66	0.849	0.544
End	8.65 ± 1	7.56 ± 1.41	**0.001**	**0.000**
*P*3	**0.001**	0.520		
Sodium				
Baseline	4.12 ± 2.76	5.80 ± 3.44	0.040	0.028
End	6.97 ± 2.54	4.61 ± 3.15	**0.002**	**0.024**
*P*3	**0.000**	0.031		
SoFAAS				
Baseline	17.20 ± 2.12	16.66 ± 1.97	0.211	0.487
End	19.10 ± 1.2 5	17.76 ± 1.83	**0.002**	**0.002**
*P*3	**0.000**	0.051		
Total score				
Baseline	60.58 ± 6.31	62.05 ± 5.7	0.350	0.283
End	83.34 ± 5.12	63.44 ± 7.49	**0.000**	**0.000**
*P*3	**0.000**	0.203		

SoFAAS.

Calories from solid fats, alcoholic beverages, and added sugars.

Data were expressed as mean ± SD.

*P*1 resulted from independent sample *t*-test; *P*2 resulted from ANCONA test (adjustment for age and energy intake); and *P*3 resulted from paired sample *t*-test.

**Table 4:** Component relative intake of HEI in the study groups at the baseline and end of study.

Components of HEI	Educated group (*n* = 30)	Noneducated group (*n* = 30)	*P*1	*P*2
Total fruit (cup per 1000 kcal)				
Baseline	0.53 ± 0.300	0.51 ± 0.19	0.246	0.689
End	1.53 ± 0.62	0.64 ± 0.24	**0.000**	**0.000**
*P*3	**0.000**	0.084		
Whole fruit (cup per 1000 kcal)				
Baseline	0.25 ± 0.12	0.18 ± 0.09	0.235	0.315
End	0.43 ± 0.27	0.20 ± 0.29	**0.000**	**0.000**
*P*3	**0.012**	0.205		
Total vegetables (cup per 1000 kcal)				
Baseline	0.73 ± 0.33	0.77 ± 0.25	0.565	0.339
End	1.31 ± 0.32	0.76 ± 0.36	**0.000**	**0.000**
*P*3	**0.000**	0.775		
Dark green and orange vegetables and legumes (cup per 1000 kcal)				
Baseline	0.12 ± 0.12	0.13 ± 0.10	0.706	0.6 53
End	0.59 ± 0.30	0.13 ± 0.09	**0.000**	**0.000**
*P*3	**0.000**	0.998		
Total grain (oz per 1000 kcal)				
Baseline	5.92 ± 0.91	6.87 ± 0.89	0.551	0.004
End	7.30 ± 0.72	6.61 ± 1.26	**0.000**	**0.000**
*P*3	**0.049**	0.063		
Whole grain (oz per 1000 kcal)				
Baseline	0.32 ± 0.07	0.62 ± 0.16	0.000	0.000
End	0.85 ± 0.42	0.28 ± 1.04	**0.000**	**0.000**
*P*3	**0.000**	0.256		
Milk (cup per 1000 kcal)				
Baseline	0.11 ± 0.08	0.10 ± 0.04	0.138	0.103
End	0.61 ± 0.244	0.08 ± 0.06	**0.000**	**0.000**
*P*3	**0.000**	0.242		
Meet and beans (oz per 1000 kcal)				
Baseline	1.62 ± 0.52	1.66 ± 0.73	0.613	0.591
End	1.76 ± 0.56	1.48 ± 0.50	**0.030**	**0.025**
*P*3	0.318	0.130		
Oils (gr per 1000 kcal)				
Baseline	9.81 ± 4.74	11.12 ± 2.42	0.553	0.127
End	7.13 ± 2.32	13.80 ± 3.75	**0.000**	**0.000**
*P*3	**0.009**	0.000		
Saturated fat (% of energy)				
Baseline	12.85 ± 2.13	13.47 ± 2.12	0.268	0.419
End	8.57 ± 2.02	13.31 ± 1.80	**0.000**	**0.000**
*P*3	**0.000**	0.614		
Sodium (gr per 1000 kcal)				
Baseline	1.82 ± 1.20	1.64 ± 1.09	0.554	0.356
End	1.02 ± 0.71	1.72 ± 0.97	**0.011**	**0.025**
*P*3	**0.000**	0.341		
SoFAAS (% of energy)				
Baseline	4.84 ± 1.50	6.72 ± 2.32	0.001	0.013
End	5.17 ± 2.07	7.88 ± 1.83	**0.000**	**0.000**
*P*3	0.432	0.044		

Data were expressed as mean ± SD.

*P*1 resulted from independent sample *t*-test; *P*2 resulted from ANCONA test (adjustment for age and energy intake); and *P*3 resulted from paired sample *t*-test.

**Table 5:** Serum levels of inflammatory markers in the study groups at the baseline and end of study.

Variables	Educated group (*n* = 30)	Noneducated group (*n* = 30)	*P*1	*P*2
Adiponectin				
Baseline	8.52 ± 2.28	8.10 ± 3.74	0.609	0.995
End	11.72 ± 4.24	7.26 ± 3.87	**0.013**	**0.035**
*P*3	**0.027**	0.330		
TNF-*α*				
Baseline	10.05 ± 4.60	9.86 ± 3.42	0.858	0.809
End	7.91 ± 2.46	9.65 ± 3.85	**0.044**	**0.011**
*P*3	**0.031**	0.605		
Hs-CRP				
Baseline	7.50 ± 3.01	7.00 ± 3.43	0.561	0.535
End	5.12 ± 2.1	7.41 ± 2.96	**0.021**	**0.039**
*P*3	**0.019**	0.271		

Data were expressed as mean ± SD.

*P*1 resulted from independent sample *t*-test; *P*2 resulted from ANCONA test (adjustment for age, weight and energy intake); and *P*3 resulted from paired sample *t*-test.

**Table 6:** Healthy eating index components and standards for scoring.

Component	Maximum points	Standard for maximum score	Standard for minimum score of zero
Total fruit (includes 100% juice)	5	≥0.8 cup/1000 kcal	No fruit
Whole fruit (not juice)	5	≥0.4 cup/1000 kcal	No whole fruit
Total vegetables	5	≥1.1 cups/1000 kcal	No vegetables
Dark green and orange vegetables and legumes	5	≥0.4 cup/1000 kcal	No dark green or orange vegetables or legumes
Total grains	5	≥3.0 cups/1000 kcal	No grains
Whole grains	5	≥1.5 oz/1000 kcal	No whole grains
Milk	10	≥1.3 cups/1000 kcal	No milk
Meat and beans	10	≥2.5 oz/1000 kcal	No meat or beans
Oils	10	≥12 grams/1000 kcal	No oil
Saturated fat	10	≤7% of energy	≥15% of energy
Sodium	10	≤0.7 gram/1000 kcal	≥2.0 grams/1000 kcal
Calories from solid fat, alcohol, and added sugar (SoFAAS)	20	≤20% of energy	≥50% of energy
